# Carbapenem-Resistant *Enterobacteriaceae* in Children, United States, 1999–2012

**DOI:** 10.3201/eid2111.150548

**Published:** 2015-11

**Authors:** Latania K. Logan, John P. Renschler, Sumanth Gandra, Robert A. Weinstein, Ramanan Laxminarayan, for the Centers for Disease Control, Prevention Epicenters Program

**Affiliations:** Rush University Medical Center, Chicago, Illinois, USA (L.K. Logan, R.A. Weinstein);; John H. Stroger, Jr. Hospital of Cook County, Chicago (L.K. Logan, R.A. Weinstein);; Center for Disease Dynamics, Economics and Policy, Washington, DC, USA (J.P. Renschler, S. Gandra, R. Laxminarayan);; Public Health Foundation of India, New Delhi, India (R. Laxminarayan);; Princeton University, Princeton, New Jersey, USA (R. Laxminarayan)

**Keywords:** carbapenem-resistant Enterobacteriaceae, CRE, bacteria, antimicrobial resistance, antibacterial agents, children, epidemiology, infections, β-lactamases, Surveillance Network–USA database, United States

## Abstract

The prevalence of carbapenem-resistant *Enterobacteriaceae* (CRE) infections is increasing in the United States. However, few studies have addressed their epidemiology in children. To phenotypically identify CRE isolates cultured from patients 1–17 years of age, we used antimicrobial susceptibilities of *Enterobacteriaceae* reported to 300 laboratories participating in The Surveillance Network–USA database during January 1999–July 2012. Of 316,253 isolates analyzed, 266 (0.08%) were identified as CRE. CRE infection rate increases were highest for *Enterobacter* species, blood culture isolates, and isolates from intensive care units, increasing from 0.0% in 1999–2000 to 5.2%, 4.5%, and 3.2%, respectively, in 2011–2012. CRE occurrence in children is increasing but remains low and is less common than that for extended-spectrum β-lactamase–producing *Enterobacteriaceae*. The molecular characterization of CRE isolates from children and clinical epidemiology of infection are essential for development of effective prevention strategies.

## Abstract 2

Infection rates have increased in all age groups and settings nationally.

Gram-negative bacteria belonging to the family *Enterobacteriaceae* are major causes of health care−acquired and community-acquired infections. In the past 3 decades, antimicrobial drug resistance in this family of bacteria has increased dramatically, specifically because of enzymes that hydrolyze broad-spectrum β-lactam antimicrobial drugs (*1*,*2*). Genes encoding AmpC cephalosporinases (AmpC) may be chromosomal or plasmid-based in origin, whereas genes encoding extended-spectrum β-lactamases (ESBLs) are most often carried on mobile genetic elements, such as plasmids or transposons, and cause resistance to all β-lactams except carbapenems and cephamycins (*1*–*4*). However, ESBLs and AmpC can confer carbapenem resistance when associated with alteration or loss of porins, a family of proteins on the outer membrane of gram-negative bacteria (*2*,*5*).

In recent years, the rapid global spread of carbapenem-resistant *Enterobacteriaceae* (CRE) has been facilitated by mobile genetic elements harboring genes encoding for carbapenemases, such as *Klebsiella pneumoniae* carbapenemase (KPC) and metallo-β-lactamases (*6*,*7*). More recently, oxacillinase 48 (OXA-48)–producing *Enterobacteriaceae* have emerged in the United States, adding to major increases in CRE infections (*8*). Carbapenem-resistant organisms often carry additional plasmid-borne genes against other antimicrobial drug classes, rendering them multidrug resistant (MDR).

Few, if any, antimicrobial drugs are able to treat these infections, and their prevalence is increasing in the United States, including in children (*9*). The National Healthcare Safety Network of the US Centers for Disease Control and Prevention (CDC) reported that the proportion of *Enterobacteriaceae* that were carbapenem-resistant increased from 1.2% in 2001 to 4.2% in 2011, and that in 2012, 4.6% of acute-care hospitals reported >1 CRE hospital-acquired infection (*10*). In a 2013 Threat Report on Antimicrobial Resistance, the CDC prioritized CRE as an urgent threat (the highest level), requiring concerted commitment and action, and noted that ≈50% of hospitalized patients with bloodstream infection caused by CRE die from the infection (*10*,*11*).

Despite this increased attention for CRE in the United States (*6*,*12*–*14*), limited data are available on the epidemiology of these infections in children (*9*,*15*,*16*). In this study, our primary objective was to describe the national and regional epidemiology of CRE in children in the United States.

## Methods

Antimicrobial drug susceptibility data were obtained from The Surveillance Network (TSN) database−USA (Eurofin-Medinet, Herndon, VA, USA). This database has been used to characterize national antimicrobial drug susceptibility trends (*14*,*17*–*19*). The network includes ≈300 clinical microbiology laboratories that serve one or more patient care facilities. Laboratories included in the network were selected to be representative of hospitals in each of the 9 US Census Bureau regional divisions. To be included in the TSN database, the laboratories must submit results from all routine antimicrobial drug susceptibility testing performed on site. Categorical result interpretations are based on the Clinical Laboratory Standards Institute (http://clsi.org/) criteria adopted by the reporting facilities at the time of testing and reflect susceptibilities as reported to clinicians. The data are electronically validated and merged into a central TSN database.

The database includes records with the following information: identified organism; tested drug and susceptibility result: susceptible, intermediate resistance, or resistant; source of the isolate: blood, urine, wound, lower respiratory tract, or other (upper respiratory tract and skin cultures); patient characteristics: age, sex; the health care setting where the patient sample was collected: outpatient (ambulatory), inpatient intensive care unit (ICU), inpatient (non-ICU), and long-term care settings; the geographic location of the laboratory where the specimen was tested; and the date of the drug susceptibility test.

Our analysis considered relevant isolates obtained from all children (age range 1−17 years) in outpatient (ambulatory), inpatient ICU, inpatient non-ICU, and long-term care settings during January 1, 1999−June 30, 2012. The included pathogens were *Escherichia coli*, *K. pneumoniae*, *Proteus mirabilis*, *Enterobacter cloacae*, *E. aerogenes*, *Citrobacter freundii*, *C. koseri*, and *Serratia marcescens*. *K. oxytoca* and *Providencia* species were not included in the TSN database. A separate analysis was performed on isolates from infants (age <1 year) because data were only available from 2010 onwards.

We defined the CRE phenotype by using CDC criteria to include relevant isolates that were resistant to all third-generation cephalosporins (ceftriaxone, cefotaxime, or ceftazidime), and nonsusceptible to >1 carbapenem (ertapenem, meropenem, imipenem, or doripenem) (*20*). Isolates that were not tested against all 3 third-generation cephalosporins were still classified as CRE if they were resistant against all tested third-generation cephalosporins. For bacteria with intrinsic imipenem nonsusceptibility (*P. mirabilis*, *Providencia* spp.), the CRE criteria required nonsusceptibility to meropenem, doripenem, or ertapenem (*20*).

Data were filtered to retain isolates that were tested against >1 third-generation cephalosporin and >1 carbapenem of those considered for the CRE phenotype. When duplicate records (with same identification number, drug susceptibility test, and source location) existed, the first record was kept and the other records were discarded. The frequency of the CRE phenotype is reported as the proportion of positive isolates of all tested isolates included in the analysis. Individual susceptibility results were stratified by location (ICU, inpatient non-ICU, and outpatient); patient age (1–5, 6–12, and 13–17 years); patient sex, isolate source (blood, urine, wound, lower respiratory tract, and other); 2-year intervals; and geographic region on the basis of the location of the laboratory (West, Northeast, South Atlantic, South Central, East North Central, and West North Central). These 6 regions correspond to the 4 US Census regions (West, Northeast, South, Midwest). The South and Midwest regions were split (into South Central and South Atlantic, and East and West North Central, respectively) to achieve a more even regional distribution of isolates.

The χ^2^ (Cochran−Armitage) test for linear trend was used to test the significance of 2-year trends. A quadratic term was added to test for a nonlinear shape of the trend. If the parameter estimate for the square of the time variable was significant and positive (negative) (p<0.05), that implied that the trend was nonlinear and the frequency of resistance was changing at an increasing (decreasing) rate. Susceptibility patterns of CRE isolates to other antimicrobial drugs were also assessed. Data were analyzed by using the R statistical software environment (*21*).

## Results

Of the 438,600 isolates from children corresponding to pathogens of interest during 1999−2012, a total of 316,253 (72.1%) met the inclusion criteria that they had been tested against >1 third-generation cephalosporin and >1 carbapenem of those considered for the CRE phenotype. Of these 316,253 isolates analyzed, 266 (0.08%) met the CRE criteria (Table 1). The median age of children for all analyzed isolates was 8 years; 120,500 (38.1%) of the isolates were from children 1–5 years of age, 100,198 (31.7%) were from children 6–12 years of age, and 95,555 (30.2%) were from children 13–17 years of age. When we considered only CRE isolates, the age distribution was skewed toward younger patients (median age 4 years), and 145 (54.5%) of isolates were from children 1–5 years of age.

**Table 1:** Characteristics of *Enterobacteriaceae* isolates and children from which they were isolated, The Surveillance Network–USA database, 1999–2012*

Characteristic	No. (%) isolates analyzed, N = 316,253	No. (%) CRE isolates analyzed, n = 266	% CRE, 266/316,253 (0.084)	Met inclusion criteria,† 316,253/438,600 (72.11)
Organism				
* Escherichia coli*	23,9274 (75.66)	58 (21.80)	0.02	70.60
* Klebsiella pneumoniae*	23,442 (7.41)	83 (31.20)	0.35	76.91
* Proteus mirabilis*	19,506 (6.17)	2 (0.75)	0.01	71.35
* Enterobacter* species‡	17,215 (5.44)	98 (36.84)	0.57	80.84
* Serratia marcescens*	10,086 (3.19)	17 (6.39)	0.17	85.77
* Citrobacter* species§	6,730 (2.13)	8 (3.01)	0.12	76.42
Health care setting				
Outpatient	245,257 (77.55)	89 (33.46)	0.04	71.28
Inpatient	53,832 (17.02)	116 (43.61)	0.22	71.71
Inpatient–ICU	10,048 (3.18)	55 (20.68)	0.55	88.12
Unknown	6,041 (1.91)	5 (1.88)	0.08	88.09
Nursing home	1,075 (0.34)	1 (0.38)	0.09	90.34
Isolate source				
Urine	265,690 (84.01)	85 (31.95)	0.03	70.30
Wound	23,269 (7.36)	66 (24.81)	0.28	80.16
Lower respiratory tract	14,400 (4.55)	74 (27.82)	0.51	86.64
Blood	8,605 (2.72)	37 (13.91)	0.43	87.56
Other¶	4,289 (1.36)	4 (1.50)	0.09	82.91
Age group, y				
1–5	120,500 (38.10)	145 (54.51)	0.12	72.43
6–12	100,198 (31.68)	63 (23.68)	0.06	71.68
13–17	95,555 (30.21)	58 (21.80)	0.06	72.14
Sex				
F	255,181 (80.69)	154 (57.89)	0.06	70.49
M	56,105 (17.74)	105 (39.47)	0.19	78.76
Unknown	4,967 (1.57)	7 (2.63)	0.14	5.37
Region				
West	78,795 (24.92)	47 (17.67)	0.06	73.62
South Atlantic	69,066 (21.84)	53 (19.92)	0.08	78.97
East North Central	57,846 (18.29)	18 (6.77)	0.03	56.13
South Central	44,414 (14.04)	28 (10.53)	0.06	82.22
North East	41,892 (13.25)	63 (23.68%)	0.15	71.35
West North Central	24,240 (7.66)	57 (21.43)	0.24	67.49

*Data for patients <1 year of age were not available for all years and excluded from analysis. CRE, carbapenem-resistant *Enterobacteriaceae*. CRE is defined as resistance to all tested third-generation cephalosporins (ceftriaxone, cefotaxime, or ceftazidime), and nonsusceptiblity to >1 carbapenem (ertapenem, imipenem, meropenem, or doripenem). For bacteria with intrinsic imipenem nonsusceptibility (*P. mirabilis*), the CRE criteria required nonsusceptibility to >2 of the carbapenems listed. ICU, intensive care unit. †Isolates were tested against >1 third-generation cephalosporin and >1 carbapenem of those considered for the CRE phenotype. ‡*E. aerogenes* and *E. cloacae*. §*C. freundii* and *C. koseri*. ¶Includes upper respiratory tract and skin cultures.

For all analyzed isolates from children, 255,181 (80.7%) were from female patients, and for the subset of CRE isolates, 154 (57.9%) were from female patients (Table 1). When we categorized all isolates by organism, isolate source, and health care setting, we found that most (239,274 [75.7%]) were *E. coli*, from urinary sources (265,690 [84%]), and from the outpatient setting (245,257 [77.6%]) (Table 1). However, among CRE isolates, the largest number of isolates were *Enterobacter* species (98 [36.8%]), from urinary sources (85 [31.9%]), and from the inpatient non-ICU setting (116 [43.6%]) (Table 1). Of the 6 geographic regions in the dataset, the largest number of isolates was from the West (78,795 [24.9%]), and for CRE isolates, the highest number of isolates was from the Northeast (63 [23.7%]) (Table 1).

**Figure 1:**
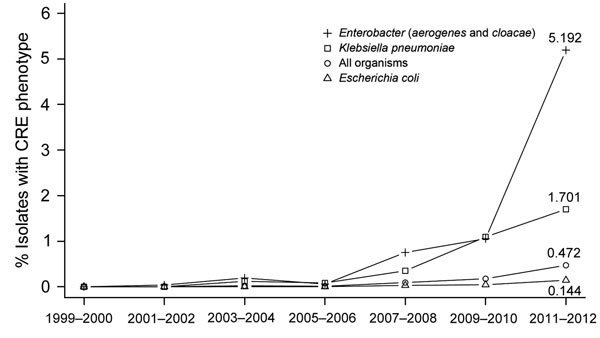
National trends in prevalence of carbapenem-resistant *Enterobacteriaceae* (CRE) isolates from children, The Surveillance Network−USA database, 1999–2012. Markers show the percentage of isolates that belonged to the resistance phenotype for each 2-year period. Data for patients <1 year of age were not available for all years and were excluded from this analysis. The All Organisms trend encompasses *Escherichia coli*, *Klebsiella pneumoniae*, *Proteus mirabilis*, *Enterobacter cloacae*, *E. aerogenes*, *Citrobacter freundii*, *C. koseri*, and *Serratia marcescens*. Each trend had a significant positive quadratic term: All Organisms (p = 1.3 × 10^−40^), *E. aerogenes* and *E. cloacae* (p = 1.4 × 10^−29^), *K. pneumoniae* (p = 6.6 × 10^−11^), and *E. coli* (p = 2.4 × 10^−11^). Trends for *C. freundii* and *C. koseri*, *S. marcescens*, and *P. mirabilis* are not shown but they all had significant positive quadratic terms (p = 0.0006; p = 0.002; and p = 1.0 × 10^−7^).

**Figure 2:**
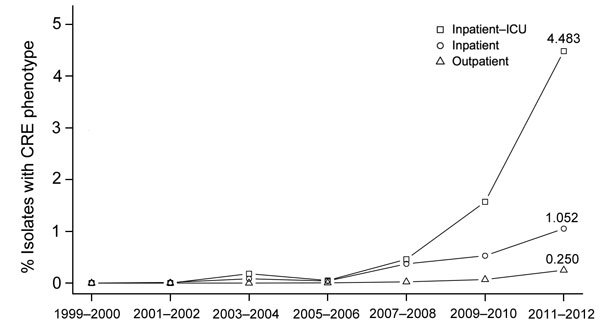
Prevalence of carbapenem-resistant *Enterobacteriaceae* (CRE) isolates from children by health care setting, The Surveillance Network-USA database, 1999–2012. Health care setting was determined by patient location at the time a microbiological sample was collected. Data for patients <1 year of age were not available for all years and were excluded from this analysis. There was a significant positive quadratic trend for intensive care unit (ICU) (p = 1.1 × 10^−18^), outpatient (p = 8.6 × 10^−26^), and inpatient non-ICU (p = 5.0 × 10^−11^). There was no significant trend for the nursing home setting, which made up 0.34% of total isolates (Table 1).

When analyzing for linear and quadratic trends during 1999−2012, we found a significant increase (p<0.0001) in the frequency of CRE isolates (Figure 1). From the 1999–2000 period until the 2011–2012 period, the frequency of CRE isolates (across all of the included *Enterobacteriaceae*) increased from 0% to 0.47%. The greatest increases were seen among *Enterobacter* species (from 0.0% in 1999–2000 to 5.2% in 2011–2012) (Technical Appendix Table 1). Likewise, there was a major increase in CRE across the ICU, inpatient non-ICU, and outpatient health care settings; the greatest increase was seen among ICU isolates (from 0.0% in 1999–2000 to 4.5% in 2011–2012) (Figure 2).

The frequency of CRE among analyzed isolates throughout the study period was also highest among male patients, children 1–5 years of age, and blood cultures (Technical Appendix Figures 1–3). Blood and lower respiratory tract cultures showed large increases of CRE over time, increasing from 0% in 1999–2000 to 3.2% and 2.3% in 2011–2012, respectively (Technical Appendix Figure 2).

**Figure 3:**
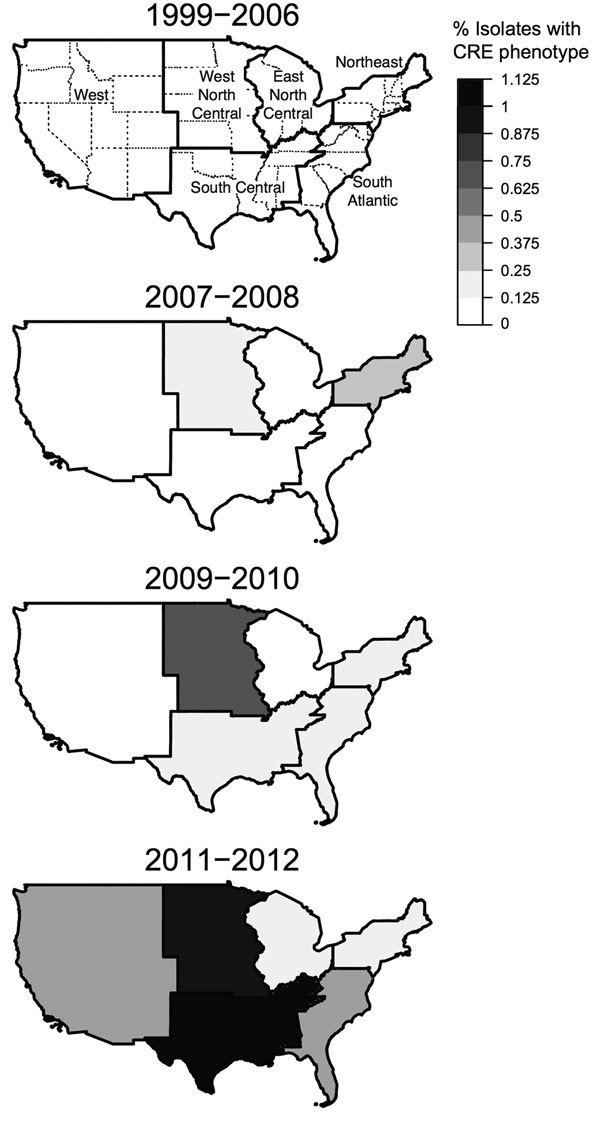
Regional trends in the prevalence of carbapenem-resistant *Enterobacteriaceae* (CRE) isolates from children, The Surveillance Network−USA database, 1999–2012. A) Percentage of isolates with CRE phenotype, 1999–2006 (0%). The 6 regions shown correspond to the 4 US Census regions (West, Northeast, South, Midwest). However, the Midwest and South regions, respectively, were split into East and West North Central and South Central and South Atlantic. Isolates from Alaska and Hawaii are included in the West region. B–D) Percentage of isolates with CRE phenotype, by 2-year period, 2007–2012. There was a significant positive quadratic trend for West (p = 4.1 × 10^−15^), South Atlantic (p = 9.4 × 10^−12^), East North Central (p = 0.0002), South Central (p = 5.2 × 10^−17^), and West North Central (p = 7.2 × 10^−8^). There was a significant linear trend for North East (p = 5.8 × 10^−8^). Data for patients <1 year of age were not available for all years and were excluded from this analysis.

Regional data are shown in Figure 3. Before 2007, the frequency of CRE was consistently low across all regions (<0.1%). In the last 2-year period (2011–2012), all regions except East North Central reached CRE prevalences >0.1%; South Central had the highest prevalence of 1.1%.

When we compared CRE counts between inpatient and outpatient settings across species, 64% (171/266) of CRE isolates were obtained from hospitalized patients (Table 2). CRE isolates were frequently resistant to additional antimicrobial drugs: 142 (54%) were resistant to trimethoprim/sulfamethoxazole, 139 (52.3%) were resistant to >1 aminoglycoside, 122 (48.2%) were resistant to ciprofloxacin, and 127 (48.3%) were multidrug resistant (nonsusceptible to >3 antimicrobial drug classes) (Table 3). CRE isolates retained the lowest phenotypic resistance to amikacin (21.3%). However, CRE isolate data did not contain information on susceptibility to other CRE treatment options, including colistin, tigecycline, polymyxin B, and fosfomycin (*22*). When the distribution of MDR isolates among CRE isolates was considered by species, we found that MDR strains were more common in *K. pneumoniae* (89.2%) and *E. coli* (50.9%) and less common in *Enterobacter* species (20.4%) (Table 4).

**Table 2:** Inpatient and outpatient CRE counts by species *Enterobacteriaceae*, The Surveillance Network–USA database, 1999–2012*

Organism	Outpatient		Inpatient and inpatient–ICU

No. (%) isolates analyzed, n = 245,257	No. (%) CRE isolates analyzed, n = 89	No. (%) isolates analyzed, n = 63,880	No. (%) CRE isolates analyzed, n = 171
*Escherichia coli*	197,807 (80.65)	27 (29.35)		36,139 (56.57)	31 (18.13)
*Proteus mirabilis*	15,738 (6.42)	2 (2.17)		8,747 (13.69)	56 (32.75)
*Klebsiella pneumoniae*	14,115 (5.76)	22 (23.91)		8,620 (13.49)	70 (40.94)
*Enterobacter* species†	8,225 (3.35)	27 (29.35)		4,907 (7.68)	8 (4.68)
*Serratia marcescens*	4,879 (1.99)	9 (9.78)		3,364 (5.27)	0
*Citrobacter* species‡	4,493 (1.83)	2 (2.17)		2,103 (3.29)	6 (3.51)

*CRE, carbapenem-resistant *Enterobacteriaceae*. CRE is defined as resistance to all tested third-generation cephalosporins (ceftriaxone, cefotaxime, or ceftazidime), and nonsusceptiblity to >1 carbapenem (ertapenem, imipenem, meropenem, or doripenem). For bacteria with intrinsic imipenem nonsusceptibility (*P. mirabilis*), the CRE criteria required nonsusceptibility to >2 of the carbapenems listed. ICU, intensive care unit. †*E. aerogenes* and *E. cloacae*. ‡*C. freundii* and *C. koseri*.

**Table 3:** Co-resistance of 266 carbapenem-resistant *Enterobacteriaceae* isolates, The Surveillance Network–USA Database 1999–2012*

Drug class or drug	No. nonsusceptible/no. tested (%)
Aminoglycosides	139/266 (52.26)
Gentamicin	108/265 (40.75)
Tobramycin	116/235 (49.36)
Amikacin	49/230 (21.30)
β-lactam/β-lactamase inhibitors	236/249 (94.78)
Ampicillin/sulbactam†	188/194 (96.91)
Piperacillin/tazobactam	201/231 (87.01)
Cefepime	125/241 (51.87)
Ciprofloxacin	122/253 (48.22)
Trimethoprim/sulfamethoxazole	142/263 (53.99)
Multidrug resistant‡	127/263 (48.29)

*Rows showing drug classes (aminoglycosides, β-lactam/β-lactamase inhibitors) indicate number of isolates that were tested against >1 drug listed in the class and the number of isolates that were nonsusceptible to >1 drug listed in the class. Tigecycline susceptibility test results were not recorded in the database. Polymyxin B and colistin susceptibility test results were recorded in the database, but none of the carbapenem-resistant *Enterobacteriaceae* (CRE) isolates were tested against those drugs. Only 1 CRE-positive isolate was tested against fosfomycin. CRE is defined as resistance to all tested third-generation cephalosporins (ceftriaxone, cefotaxime, or ceftazidime), and nonsusceptiblity to >1 carbapenem (ertapenem, imipenem, meropenem, or doripenem). For bacteria with intrinsic imipenem nonsusceptibility (*P. mirabilis*), the CRE criteria required nonsusceptibility to >2 of the carbapenems listed. †*Citrobacter* (n = 8) and *Enterobacter* (n = 98) species are intrinsically resistant (*22*) to ampicillin/sulbactam. However, they have been included in the table. ‡These CRE isolates were nonsusceptible to >1 drug from each of the following 3 drug classes: aminoglycosides (gentamicin, tobramycin, amikacin), β-lactams (ampicillin/sulbactam, piperacillin/tazobactam), fluoroquinolones (ciprofloxacin), and trimethoprim/sulfamethoxazole.

**Table 4:** CRE and non-CRE multidrug-resistant isolates by species, The Surveillance Network–USA database, 1999–2012*

Organism	Non-CRE, no. MDR†/no tested‡ (%)	CRE, no. MDR†/no. tested‡ (%)
All species	11,718/314,573 (3.73)	127/263 (48.29)
*Escherichia coli*	8,402/238,709 (3.52)	29/57 (50.88)
*Klebsiella pneumoniae*	1,223/23,263 (5.26)	74/83 (89.16)
*Proteus mirabilis*	390/19,458 (2.00)	0/2 (0.00)
*Enterobacter* species§	775/16,867 (4.59)	20/98 (20.41)
*Serratia marcescens*	513/9,629 (5.33)	4/15 (26.67)
*Citrobacter* species¶	415/6,647 (6.24)	0/8 (0.00)

*CRE, carbapenem-resistant *Enterobacteriaceae*. CRE is defined as resistance to all tested third-generation cephalosporins (ceftriaxone, cefotaxime, or ceftazidime), and nonsusceptiblity to >1 carbapenem (ertapenem, imipenem, meropenem, or doripenem). For bacteria with intrinsic imipenem nonsusceptibility (*P. mirabilis*), the CRE criteria required nonsusceptibility to >2 of the carbapenems listed. ICU, intensive care unit. †These isolates were nonsusceptible to >1 drug from each of the following 3 drug classes: aminoglycosides (gentamicin, tobramycin, amikacin), β-lactams (aAmpicillin/sulbactam, piperacillin/tazobactam), fluoroquinolones (ciprofloxacin), and trimethoprim/sulfamethoxazole. ‡These isolates were tested against >1 drug from each of the following 3 drug classes: aminoglycosides (gentamicin, tobramycin, amikacin}, β-lactams (ampicillin/sulbactam, piperacillin/tazobactam), fluoroquinolones (ciprofloxacin, and trimethoprim/sulfamethoxazole. §*E. aerogenes* and *E. cloacae.* ¶*C. freundii* and *C. koseri.*

CRE trends were not analyzed for isolates from children <1 year of age because of lack of data before 2010. However, data for this age group collected during 2010–2012 demonstrated resistance levels consistent with those seen in other age cohorts (Technical Appendix Table 2). Of the 8,319 isolates, 70 (0.8%) met CRE criteria. *E. aerogenes* and *E*. *cloacae* isolates represented the largest group of CRE isolates (41 [58.6%]) compared with other organisms, as did male patients (42 [60%]) compared with female patients and isolates from urinary sources (32 [45.7%]) compared with other sources.

## Discussion

In our nationally representative sample, we found that CRE in US children showed a major increase during 1999–2012, and the most substantial increases were in children 1–5 years of age, male patients, blood culture isolates, and the ICU setting. However, overall CRE occurrence in children remained low and relatively uncommon compared with ESBL-producing *Enterobacteriaceae*. The proportion of ESBL-producing *Enterobacteriaceae* from the same TSN database was much higher than that of CRE (0.47% ESBL vs. 0.08% CRE) in children (*19*).

Dissemination of KPC accounts for most of the increasing prevalence of CRE in the United States (*24*). However, in the past 5 years, other carbapenemases that are also rapidly spread by mobile genetic elements harboring genes encoding carbapenemases, such as metallo-β-lactamases (MBLs), including New Delhi MBLs, Verona integron−encoded MBLs, and IMPs (active on imipenem), as well as Class D OXA-producing enzymes (such as OXA-48), have also been reported in clinical *Enterobacteriaceae* isolates from the United States (*8*,*24*). CRE infections have been associated with high rates of illness and death (*25*).

Risk factors for CRE are well described in adults and include critical illness, immune compromise, exposure to health care, residence in long-term health care facilities, longer length of stay before infection, and prior exposure to antimicrobial drugs (*13*,*25**,**28*). However, little is known about the epidemiology of these factors for children (*9*), and published data on the prevalence of CRE in children have been scarce. Moreover, the increase in prevalence in children over the past decade has not been well described. Unlike for adults, where increases were greater than for children, we did not find that the increase in CRE in children appeared to be related to residence in long-term care facilities, because only 0.1% of CRE isolates came from this setting (*13*). However, long-term care facilities have been described as a potential risk factor for colonization in children (*16*), and it is possible that this factor was missed in our study because patient location entered in laboratory information systems might not correspond to the clinical setting in which patients ultimately received care. We observed that CRE are more commonly isolated with hospitalized patients (Table 2).

There are few treatment options for CRE infections. This armamentarium is further reduced for children and pediatric clinical data are lacking (*29*). Side effect profiles limit tigecycline use for persons <18 years of age, and the US Food and Drug Administration discourages routine use because of increased risk for death (*30*). Several questions remain about optimal pediatric dosing of polymyxins, such as colistin. Oral fosfomycin is available for the treatment of CRE cystitis; however, standard dosing guidelines are available only for older children and adolescents (*29*).

Furthermore, CRE are known to harbor additional drug-resistance genes to other antimicrobial drug classes, which may also be carried on mobile genetic elements. *K. pneumoniae* sequence type 258 strains are KPC-producing clones that harbor Tn*4401*-bearing plasmids. These clones are highly effective in plasmid transfer across bacteria and are known to carry other plasmid-based antimicrobial drug resistance genes such as those that encode resistance to trimethoprim/sulfamethoxazole, aminoglycosides, and fluoroquinolones (*6*). For *Enterobacter* species, various typing methods have been used to study clonal lineages, including pulse-field gel electrophoresis (*31*), repetitive sequence PCR (*32*), and, more recently, multilocus sequence typing (*33*) and partial sequencing of the housekeeping gene *hsp*60, which suggests that KPC-producing *Enterobacter* strains are clustered within specific genetic groups (*34*).

It has been well documented that carbapenemase-bearing plasmids frequently carry determinants of resistance to multiple drug classes (*1*,*6*,*9*). Thus, we propose that the high levels of multidrug resistance in carbapenem-resistant *K. pneumoniae* and *E. coli* isolates from children and lower levels in *Enterobacter* species argue in favor of increased prevalence of plasmid-mediated carbapenemase production as the basis for increases in carbapenem resistance in *K. pneumoniae* and *E. coli*, and increased prevalence of other mechanisms (e.g., chromosomal AmpC cephalosporinase induction/derepression or porin modification) as the basis in *Enterobacter* species.

For MDR strains, this resistance could be reflective of dissemination of the sequence type 258 clone. However, the increase in CRE in children might also be caused by additional MDR genetic clusters, because single-center molecular studies of KPC isolates from children have reported that a more polyclonal epidemiology may be responsible for dissemination of KPC in children (*16*,*35*). Among the *Enterobacter* species, MDR strains were less common when compared with *K. pneumoniae* and *E. coli*. Thus, the sensitivity of current surveillance definitions may capture nosocomial ecology and not reflect true carbapenemase production among *Enterobacter* species.

In addition, *S. marcescens* (comprising 6.4% of CRE isolates in this study) is known to harbor SME, a serine carbapenemase, as a mechanism of carbapenem resistance (*1*,*2*). Isolates with this phenotype may retain susceptibility to cefepime. Of the *S. marcescens* in this dataset, 40% were resistant to cefepime.

Although data for infants (children <1 year of age) were only available in the last 2 years of the study (2010–2012), resistance levels in infants were similar to those in other age cohorts, suggesting increases in this age group. The epidemiology of colonization and infection in this age group might differ from that of the overall pediatric population because cases have been described as being caused by vertical transmission or by other risk factors associated with neonatal ICU acquisition; however, data remain limited (*9*,*36*). Available data on colonization with MDR *Enterobacteriaceae* in pediatric patients suggest that intestinal carriage of these organisms can last for months to years in some children and that this might be associated with reinfection or potential spread to other family members (*37*,*38*).

Our study has major limitations. First, we cannot distinguish between true infection and colonization, especially among non-blood isolates. Second, because of the nature of the TSN data, it is not possible to avoid bias caused by multiple health care visits made by the same patient over the course of an infection because each time a patient is admitted, a new identification number is assigned that is used to tag any specimens obtained during that health care visit. Third, in June 2010, Clinical Laboratory Standards Institute clinical breakpoints for carbapenems against *Enterobacteriaceae* were decreased. Because MICs were not available, we ran truncated analyses that included all isolates collected before June 2010. All pediatric CRE prevalence trends were still significant. In addition, many clinical laboratories have been slow to adopt these breakpoint revisions. Fourth, because the overall numbers of CRE isolates in the study were small, resistance trends could be potentially affected by an outbreak at 1 or a small number of institutions. Fifth, molecular mechanisms of carbapenem resistance among the isolates could not be determined. Sixth, although we used CDC criteria to define the CRE phenotype, some mechanisms of resistance other than carbapenemase production might account for carbapenem resistance in isolates.

In summary, the prevalence of CRE infections is increasing among children in the United States but CRE remain relatively uncommon. Molecular characterization is necessary to determine specific CRE genotypes associated with this spread. Continued vigilance for CRE and initiation of the CDC 4 core actions to prevent antimicrobial drug resistance (*11*) should be emphasized for all patient populations, including children.

**Technical Appendix.** Additional information on carbapenem-resistant *Enterobacteriaceae* in children, United States, 1999–2012.
