# Supplementary material for: Carbapenem-Resistant Enterobacteriaceae in Children, United States, 1999–2012
Source: Emerg Infect Dis. 2015 Nov;21(11):2014–21. doi: 10.3201/eid2111.150548 (PMC4622248; doi:10.3201/eid2111.150548)
Supplement: Supplementary file 1 — Technical Appendix. Additional information on carbapenem-resistant Enterobacteriaceae in children, United States, 1999–2012. [file 15-0548-Techapp-s1.pdf]

Article DOI: http://dx.doi.org/10.3201/eid2111.150548

Carbapenem-Resistant *Enterobacteriaceae* in Children, United States, 1999–2012

Technical Appendix

Technical Appendix Table 1. Two-year CRE isolate counts by species, the Surveillance Network–USA database, 1999–2012*

Organism	1999–2000	2001–2002	2003–2004	2005–2006	2007–2008	2009–2010	2011–2012
Enterobacter species†	0	1	6	2	20	23	46
Escherichia coli	0	0	1	1	12	16	28
Klebsiella pneumoniae	0	0	5	4	14	35	25
Serratia marcescens	0	0	0	0	3	3	11
Citrobacter species‡	0	0	0	0	0	5	3
Proteus mirabilis	0	0	0	0	0	0	2

*CRE, carbapenem-resistant *Enterobacteriaceae*. CRE is defined as resistance to all tested third-generation cephalosporins (ceftriaxone, cefotaxime, or ceftazidime), and nonsusceptiblity to ≥1 carbapenem (ertapenem, imipenem, meropenem, or doripenem). For bacteria with intrinsic imipenem nonsusceptibility (*P. mirabilis*), the CRE criteria required nonsusceptibility to ≥2 of the carbapenems listed.
†*E. aerogenes* and *E. cloacae.*
‡*C. freundii* and *C. koseri.*

Technical Appendix Table 2. Characteristics of *Enterobacteriaceae* isolates and children >1 year of age from which they were isolated, The Surveillance Network–USA database, 1999–2012*

Characteristic	No. (%) isolates tested, n = 8,319	No. (%) CRE isolates tested, n = 70	No. CRE/no. tested, %, 70/8,319 (0.84%)
Organism			
Escherichia coli	4,733 (56.89)	4 (5.71)	0.08
Klebsiella pneumonia	1,302 (15.65)	17 (24.29)	1.31
Enterobacter species†	1,298 (15.60)	41 (58.57)	3.16
Serratia marcescens	448 (5.39)	4 (5.71)	0.89
Citrobacter species‡	274 (3.29)	4 (5.71)	1.46
Proteus mirabilis	264 (3.17)	0	0.00
Health care setting			
Outpatient	3,869 (46.51)	17 (24.29)	0.44
Inpatient	2,543 (30.57)	27 (38.57)	1.06
Inpatient–ICU	1,887 (22.68)	26 (37.14)	1.38
Nursing home	12 (0.14)	0	0.00
Unknown	8 (0.10)	0	0.00
Isolate source			
Urine	4,860 (58.42)	32 (45.71)	0.66
Lower respiratory tract	1,673 (20.11)	30 (42.86)	1.79
Wound	921 (11.07)	7 (10.00)	0.76
Blood	548 (6.59)	1 (1.43)	0.18
Other§	317 (3.81)	0	0.00
Sex			
F	4,197 (50.45)	28 (40.00)	0.67
M	4,106 (49.36)	42 (60.00)	1.02
Unknown	16 (0.19)	0	0.00
Region			
South Atlantic	2,754 (33.10)	21 (30.00)	0.76
West	2,015 (24.22)	14 (20.00)	0.69
South Central	1,228 (14.76)	7 (10.00)	0.57
West North Central	966 (11.61)	11 (15.71)	1.14
East North Central	755 (9.08)	16 (22.86)	2.12
North East	601 (7.22)	1 (1.43)	0.17

*CRE, carbapenem-resistant *Enterobacteriaceae*. CRE is defined as resistance to all tested third-generation cephalosporins (ceftriaxone, cefotaxime, or ceftazidime), and nonsusceptiblity to ≥1 carbapenem (ertapenem, imipenem, meropenem, or doripenem). For bacteria with intrinsic imipenem nonsusceptibility (*P. mirabilis*), the CRE criteria required nonsusceptibility to ≥2 of the carbapenems listed. ICU, intensive care unit.
†Isolates were tested against ≥1 third-generation cephalosporin and ≥1 carbapenem of those considered for the CRE phenotype.
†*E. aerogenes* and *E. cloacae*.
‡*C. freundii* and *C. koseri*.
§Includes upper respiratory tract and skin cultures.

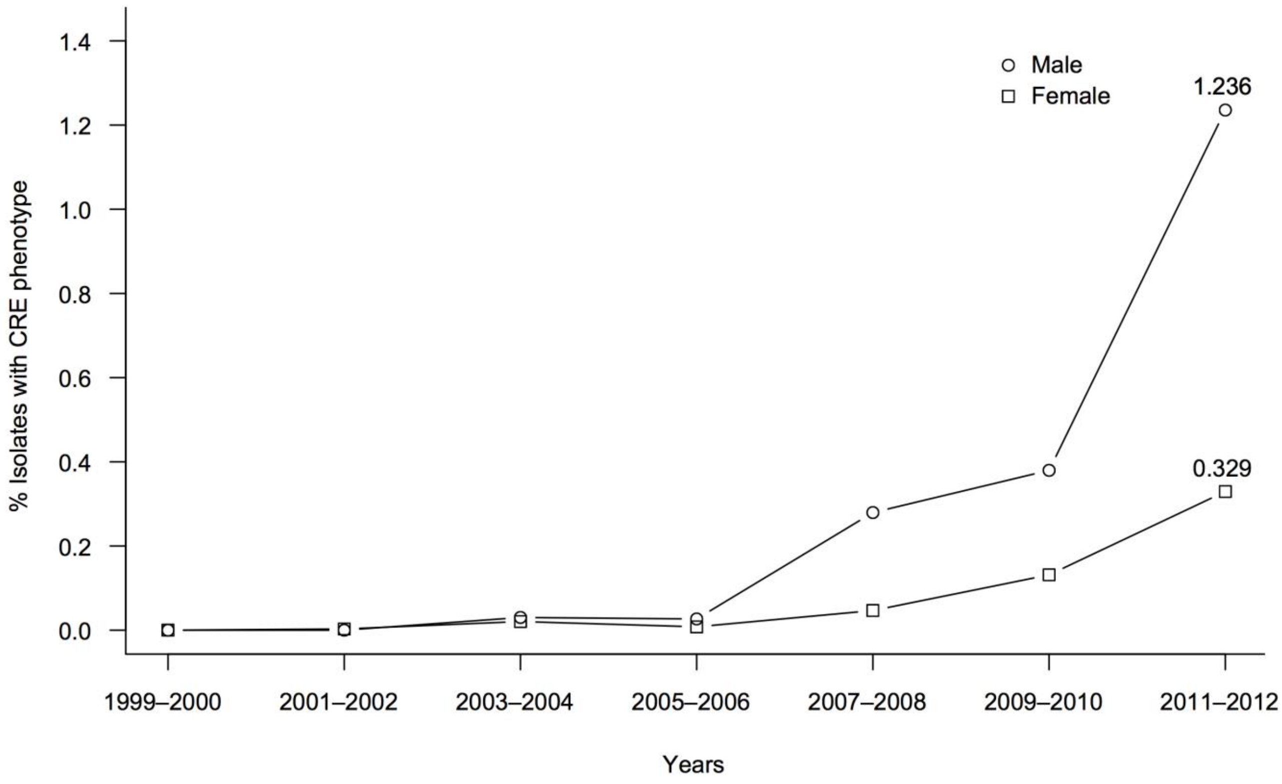

Technical Appendix Figure 1. Patient sex and national trends in prevalence of third-generation cephalosporin– and carbapenem-resistant *Enterobacteriaceae* (CRE) isolates from children, The Surveillance Network–USA database, 1999–2012. Data for patients <1 year of age were not available for all years and were excluded from analysis. There was a significant positive quadratic trend for male patients ($p = 3.4 \times 10^{-19}$) and female patients ($p = 2.5 \times 10^{-25}$).

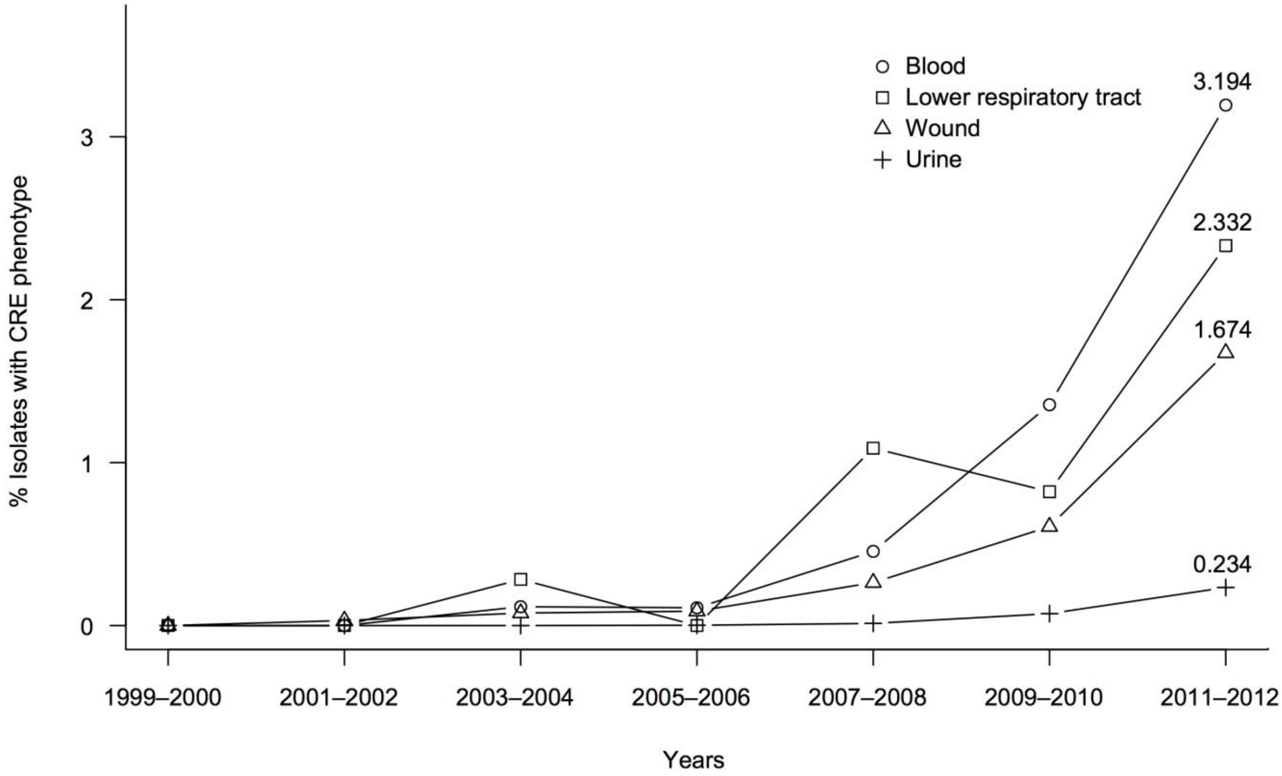

Technical Appendix Figure 2. Isolate source and national trends in prevalence of third-generation cephalosporin– and carbapenem-resistant *Enterobacteriaceae* (CRE) isolates from children, The Surveillance Network–USA database, 1999–2012. Data for patients <1 year of age were not available for all years and were excluded from analysis. There was a positive quadratic trend for urine ($p = 3.1 \times 10^{-29}$), blood ($p = 2.0 \times 10^{-10}$), wound ($p = 1.1 \times 10^{-11}$), and lower respiratory tract ($p = 7.4 \times 10^{-6}$).

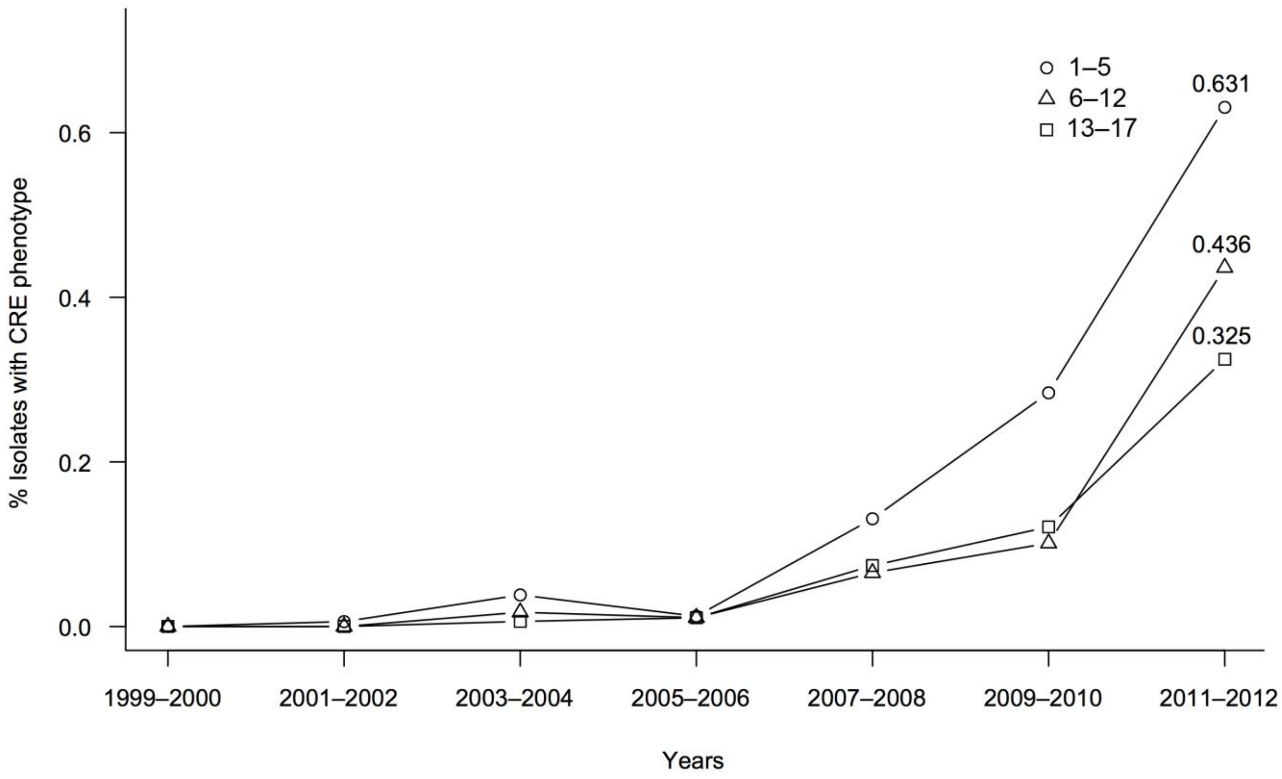

Technical Appendix Figure 3. Age group and national trends in prevalence of third-generation cephalosporin– and carbapenem-resistant *Enterobacteriaceae* (CRE) isolates from children, The Surveillance Network–USA database, 1999–2012. Data for patients <1 year of age were not available for all years and were excluded from analysis. There was a significant positive quadratic trend for ages 1–5 ($p = 1.3 \times 10^{-20}$), 6–12 ($p = 1.2 \times 10^{-14}$), and 13–17 years ($p = 1.8 \times 10^{-9}$).
